# Financial Evaluation of Different Vaccination Strategies for Controlling the Bluetongue Virus Serotype 8 Epidemic in the Netherlands in 2008

**DOI:** 10.1371/journal.pone.0019612

**Published:** 2011-05-04

**Authors:** Annet G. J. Velthuis, Monique C. M. Mourits, Helmut W. Saatkamp, Aline A. de Koeijer, Armin R. W. Elbers

**Affiliations:** 1 Business Economics, Wageningen University, Wageningen, The Netherlands; 2 Crisis Organization and Diagnostics, Department of Epidemiology, Central Veterinary Institute (CVI), part of Wageningen UR, The Netherlands; Erasmus University Rotterdam, The Netherlands

## Abstract

**Background:**

Bluetongue (BT) is a vector-borne disease of ruminants caused by bluetongue virus that is transmitted by biting midges (*Culicoides* spp.). In 2006, the introduction of BTV serotype 8 (BTV-8) caused a severe epidemic in Western and Central Europe. The principal effective veterinary measure in response to BT was believed to be vaccination accompanied by other measures such as movement restrictions and surveillance. As the number of vaccine doses available at the start of the vaccination campaign was rather uncertain, the Dutch Ministry of Agriculture, Nature and Food Quality and the Dutch agricultural industry wanted to evaluate several different vaccination strategies. This study aimed to rank eight vaccination strategies based on their efficiency (i.e. net costs in relation to prevented losses or benefits) for controlling the bluetongue virus serotype 8 epidemic in 2008.

**Methodology/Principal Findings:**

An economic model was developed that included the Dutch professional cattle, sheep and goat sectors together with the hobby farms. Strategies were evaluated based on the least cost - highest benefit frontier, the benefit-cost ratio and the total net returns. Strategy F, where all adult sheep at professional farms in the Netherlands would be vaccinated was very efficient at lowest costs, whereas strategy D, where additional to all adult sheep at professional farms also all adult cattle in the four Northern provinces would be vaccinated, was also very efficient but at a little higher costs. Strategy C, where all adult sheep and cattle at professional farms in the whole of the Netherlands would be vaccinated was also efficient but again at higher costs.

**Conclusions/Significance:**

This study demonstrates that a financial analysis differentiates between vaccination strategies and indicates important decision rules based on efficiency.

## Introduction

Bluetongue (BT) is a non-contagious, vector-borne disease of ruminants and camelids caused by bluetongue virus (BTV) that is transmitted by biting midges (*Culicoides* spp.) [1]. Animals, in particular sheep, can develop severe clinical symptoms as a result of the infection. Due to the economic damage historically associated with Bluetongue, it was listed as a notifiable disease by the World Organization for Animal Health (OIE) in the 1960's. As a consequence, the OIE standards require that all international movements of animals of susceptible species and their potential infectious products from infected countries and zones are forbidden unless they are demonstrated to be non-infected after a specified period of protection from vector attacks in an insect-proof environment [2].

In 2006, the introduction of BTV serotype 8 (BTV-8) caused a severe epidemic in Western and Central Europe [3]. Although a comprehensive set of control measures (including obligatory indoor housing, treatments with insecticides, trade restrictions like restrictions on animal movements and extra testing and controls on export animals) was put in place at the national and the European Union (EU) level in 2006, the infection reappeared after a mild winter and spring in 2007. The epidemic developed quickly over a large part of Western and Central Europe, affecting approximately 40,000 locations with ruminants [4].

Vaccination has been demonstrated to be an effective tool to control the spread of BT [5]. The European Commissioner for Health, Markos Kyprianou, stated at the EU Conference on “Vaccination Strategy against Bluetongue (16 January 2008)” that the principal, and possibly the only, effective veterinary measure in response to BT is vaccination accompanied by ancillary measures such as movement restrictions and surveillance. The scale of the epidemic in 2007 was so large that an emergency vaccination campaign was started in May and June of 2008 in several EU member states [6], [7], [8]. In some member states like Belgium, Germany and Luxembourg a mandatory vaccination campaign was initiated, in others like Britain and the Netherlands a voluntary campaign was promoted [9]. The emergency vaccination campaign against BTV-8 in 2008 was financially supported by the EU, as laid down by Decision 2008/655/EC [10].

As the number of vaccine doses available at the start of the vaccination campaign was rather uncertain, the Dutch Ministry of Agriculture, Nature and Food Quality and the Dutch agricultural industry wanted to evaluate several different vaccination strategies. This evaluation should focus on the costs and benefits for the year of vaccination It should include all financial costs and benefits of a wider farm perspective, including the affected farm sector (cattle, sheep and goats), the affected related industry and the control authorities, which are either paid by the government, animal health authorities or the farmers and firms. Therefore, the objective of this study was to rank different possible vaccination strategies based on the efficiency (i.e. net costs in relation to prevented losses or benefits) for controlling the BTV-8 epidemic in the period July 2008 to July 2009 to support animal health authority in their decision making.

## Materials and Methods

For the financial analysis the following steps were followed. First, the different vaccination strategies were defined. Second, the financial impact of a BTV-8 epidemic in 2008 (i.e. from July 2008 to July 2009) was quantified under the condition that no vaccination strategy would be applied. Third, the reduction in financial impact of the BTV-8 epidemic in 2008 due to the application of a vaccination strategy was quantified. Fourth, the costs of the defined vaccination strategies were calculated. Fifth, the different vaccination strategies were ranked based on economic criteria. And sixth, a sensitivity analysis was performed.

### Vaccination strategies evaluated

Eight vaccination strategies were determined by veterinary epidemiologists who were closely involved with the Dutch BTV-8 epidemic. They advised the Dutch government during the epidemic based on their knowledge which was obtained from on-going epidemiological research during that period and from international literature and discussion groups. During a discussion meeting the epidemiologists decided on the strategies evaluated. These strategies were considered in the decision making process and were based on the uncertain knowledge on the number of vaccine doses available in June 2008. This uncertainty forced the decision makers to think about vaccinating sub groups, e.g. differentiate in age and/or regions (e.g. the Northern part of the Netherlands was most susceptible at the end of 2007 and should be prioritized for vaccination [11]) and/or farm type (professional and/or hobby farms). The eight defined vaccination strategies are: A, Vaccination of all sheep, cattle and goats of the Netherlands; B, vaccination of all adult sheep, cattle and goats of the Netherlands; C, vaccination of all adult sheep and cattle at professional farms in the Netherlands; D, vaccination of all adult sheep at professional farms of the Netherlands and all adult cattle in the four Northern provinces (Groningen, Friesland, Drenthe and North Holland); E, vaccination of all adult cattle at professional farms in the Netherlands; F, vaccination of all adult sheep at professional farms in the Netherlands; G, vaccination of all adult cattle at professional farms in the four Northern provinces; H, vaccination of 80% of the adult sheep and cattle in the Netherlands.

Strategies A and B were based on unlimited availability of vaccine doses. Strategy C was based on the availability of three million doses. Strategies D and E were based on availability of two million doses and strategy F and G on availability of one million doses. Strategy H differs from the others. In this scenario a voluntary vaccination program is assumed with the expectation that 80% of the sheep and cattle owners would decide to vaccinate. All other programs were assumed to be obligatory with the expectation that 100% of the animals owners who should vaccinate, would vaccinate.

### Estimation of the benefits of the vaccination strategies

The economic benefit of vaccination against BTV-8 is defined as the reduction of potential damage or financial impact due to vaccination caused by a BTV-8 epidemic in the cattle, sheep and goat sectors from July 2008 to July 2009. The reduction in losses due to vaccination can be calculated based on the estimated damage in a situation where no vaccination strategy would be applied, which is indicated with scenario ‘BT2008-NV’. All vaccination scenarios and the BT2008-NV scenario take account of the same specific starting situation, which is the number of non-infected farms the winter of 2007/2008 in the Northern, Middle, and Southern part of the Netherlands. At this time the virus has already infected 94% of all cattle holdings, 70% of all sheep holdings and 47% of all goat holdings [11]. In the BT2008-NV scenario it is assumed that all non-infected farms (i.e. not infected during the epidemics of 2006 and 2007) would be infected in 2008 if no vaccination strategy would be applied, i.e. a worst case scenario. For the farms that were infected before, we assumed that a new infection in 2008 will have negligible effect based on the observation that farms infected in 2006 had negligible health problems due to BTV-8 during the epidemic of 2007.

The financial impact of the BT2008-NV epidemic is calculated using the economic model as described in Velthuis et al. [11]. The deterministic model includes all costs and benefits of affected farms (cattle, sheep and goats), affected related industries (like dairy companies, slaughterhouses or export related firms) and the control authorities. As at the time of this study no losses or benefits due to the BTV-8 epidemic were identified for dairy companies and slaughterhouses, they were not in the model and will not be presented in the results of this study.

The model calculates the net costs or financial impact of Dutch BTV-8 epidemics by integrating demographic, epidemiologic and economic data. In addition, it is compatible with the Dutch livestock production systems for cattle, sheep and goats, including the hobby holdings. Three different regions were distinguished in the model, namely North, Central and South. as numbers of farms and farm sizes varied among the regions as well as the observed BT morbidity and mortality rates. The northern region includes the provinces of Friesland, Groningen, Drenthe and North Holland, the central region comprises the provinces of Gelderland, Overijssel, Flevoland and Utrecht, and the southern region of the provinces of North Brabant, South Holland, Zeeland and Limburg.

The net costs of a BTV-8 epidemic (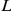) include the impact of BT on production (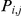) for farm type i and animal type 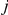, treatment of diseased animals (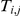), diagnostic costs (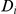), and costs of control measures adopted during the course of the epidemic, including price changes for animals and animal products due to transport restrictions (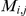):
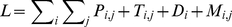
(1)Details of the calculations are given in Velthuis et al. [11], whereas in the following paragraphs only the main assumptions and changes are described.

The production effects that exhibit financial consequences (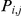) included mortality, early culling, reduced milk production, weight loss, no gestations, postponed gestations, abortions, less fertile sheep rams, lower birth weights, and stillbirths.

The treatment cost (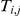) included costs of the veterinary medicines and application materials. Costs for the veterinarian were not included in the treatment costs but in the costs for diagnosis. This is because most sick animals are treated during the (first) visit where BTV-8 is diagnosed. Furthermore, it is assumed that subsequent treatments were applied by farmers where it is assumed that there are no opportunity costs for this labour. To relieve the suffering and to prevent secondary infections as a result of reduced immunity, part of the BTV-8 diseased animals were treated with pain killers, antibiotics or corticosteroids. If a dairy cow was treated with antibiotics the calculations also included additional losses due to the fact that milk cannot be delivered for some period.

Diagnostic costs (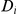) included the costs of veterinary labour (state and/or private veterinarian), sampling materials and test costs (including the submission costs to the lab). The official BTV-8 diagnosis for 2008 was based exclusively on clinical inspections by private veterinarians, like it was defined from October 2007. However, a number of samples was still submitted to the lab and were therefore included in the calculations.

The control measures for livestock farms in various control zones around infected farms that were applied in 2007 were assumed to be applied in 2008 too. These measures include treatment of animals, stables and vehicles for animal transport with insecticides, extra testing and control of animals for export, and restrictions on animal movements. The losses due to restrictions on animal movement were considered to be equivalent to the price changes of animals and animal products because they affect either the supply or demand in a region which consequently leads to price changes. An autoregressive integrated moving average model was used to test if changes in the National average monthly prices are related to the estimated number of farms in restriction zones. For this, monthly data of the years 2004, 2005 and 2006 were used to correct for normal seasonal influences. Next, the results have been discussed with the market experts from different sectors to verify their credibility. Based on this procedure it was concluded that prices were not changed due to the BTV8 epidemic, except for the export heifer prices. The latter price increase was for 50% attributed to the decreased supply of export heifers from BTV8 infected EU member states and for 50% attributed to an increased worldwide demand for dairy milk and therefore cattle, so 50% of the price increase was included in the calculations.

The inputs for the calculation of the possible losses due to a BT2008-NV epidemic were in most cases the same as assumed for the BTV-8 epidemic in 2007 [11]. Only the epidemiological inputs for mortality and morbidity and the number of infected farms differed. The new BT2008-NV inputs were based on the opinion of five BT experts: four epidemiologists and one veterinarian closely involved with the Dutch BTV-8 epidemic and related research. The experts have attended a discussion meeting and agreed on the new inputs and assumptions of the model with regard to the BT2008-NV epidemic. The inputs are given in Table 1.

**Table 1:** Input for the cost calculations regarding BTV-8 vaccination strategies.

		Input parameters											
		For all	cattle farms			sheep farms					goats farms		
Var.	Description	Farms	dairy	veal	others	dairy	herding	breeding	fattening	hobby	dairy	fattening	hobby
*n_j_*	# farms		22,301	3,174	10,771	30	40	10,432	2,000	51,881	351	45	74,824
*d_k_*	# doses per animal		2	2	2	1	1	1	1	1	1	1	1
*Vet_1_*	Call out charge vet €/visit^1^	20.58											
*h_j_*	# hours to vaccinate a farm^1^		1	2	1	1	1	1	1	0.5	1	1	0.5
*Vet_2_*	Hourly rate vet €/hour^1^	116.17											
*na_j_*	# animals to be vaccinated												
	all animals		113	266	35	245	1,044	88	58	6	612	297	4
	only adults		64	0	18	240	448	51	0	4	447	0	3
*M*	Materials costs €/animal	0.02											
*V*	Vaccine price €/dosage^1^	0.40											
*R*	Registration costs €/animal	0.05											

1 for the calculation of the full costs, i.e. without the EU compensation.

The expected number of infected holdings in the baseline scenario BT2008-NV was assumed to be equal to the number of non-infected farms in 2007. This assumption is based on the fact that the farm level seroprevalence was very high at the end of 2007 [11] and assuming that infected animals are not susceptible for the infection in the next year. Both assumptions indicate that the number of susceptible animals in 2008 was very low (Table 2). The mortality and morbidity rates in the northern region during the BT2008-NV epidemic were assumed to equal the rates in the central region in 2007. This because the rates in 2007 were higher in the central region compared to the northern and southern regions, suggesting that the rates increase with the number of infected farms and animals in the region. The mortality and morbidity rates in the central region in 2008 were assumed to be 85% of the rates in the central region in 2007. This reduction is assumed since the number of new infected farms in that region in 2008 would be lower than in 2007. Finally, the mortality and morbidity rates in the southern region in 2008 were assumed to be 50% of the rates in the southern region in 2007. This reduction reflects also the reduction in new infected farms in 2008 compared to 2007.

**Table 2:** Input for the calculation of losses of a “BTV-8 2008 epidemic without vaccination.”

	Cattle			Sheep			Goats
Description	North	Middle	South	North	Middle	South	-
% infected farms	17.30	0.56	2.68	30.39	30.39	30.39	
Estimated # Infected farms	1,505	98	188	7,165	9,286	3,114	40
Mortality rate A^1^ (#/100 animal months)	0.225	0.196	0.085	1.233	1.048	0.799	0.000
B (#/100 animal months)	0.256	0.218	0.200	1.233	1.048	0.799	0.000
Morbidity rate A (#/100 animal months)	6.480	5.508	3.223	6.484	5.511	3.997	1.300
B (#/100 animal months)	0.808	0.687	0.514	6.484	5.511	3.997	1.300

1 Mortality rates A and B are based on different estimates for the epidemic in 2007 (see Velthuis, et.al., 2010).

### Epidemiological scenarios ES1 and ES2

Different estimates of mortality and morbidity rates for cattle during the 2007 epidemic exist and therefore two different epidemiological scenarios were assumed in this study: ES1 and ES2. The rates for scenario ES1 were based on a longitudinal study of 585 BTV-8 confirmed-infected cattle farms [4], whereas the rates in scenario ES2 are based on a longitudinal study of 72 dairy farms [12], [13].

### Effect of vaccination

The economic benefit of vaccination against BTV-8 is defined as the reduction of the financial impact due to vaccination caused by a BTV-8 epidemic from July 2008 to July 2009 compared to the baseline scenario BT2008-NV. This reduction is due to the effect of the vaccine on the health state of the vaccinated animals, with the consequence that less control measures are needed to control the epidemic. We assumed that animals were vaccinated a few weeks before being exposed to BTV-8 so that the effect of vaccination was maximal. Vaccination will reduce the mortality and morbidity rates to zero because it is assumed that vaccination will induce full clinical protection [14], [15]. So, vaccinated animals will not get diseased or die as a result of BT infection. As a consequence, the production losses due to a BTV-8 infection will be absent and no treatments are needed to help sick animals recover. We assumed that vaccination does not induce negative health effects to the animals [7], [16]. Long term effects of vaccination beyond 2008, like i.e. the benefit of elimination and consequently a potential change of the countries health status, were behind the scope of this research. It was assumed that, only costs are made for diagnosis on vaccinated farms if they were nominated to export production animals as a full protection needed to be guaranteed.

### Cost calculations of the vaccination strategies

The total costs of the defined vaccination strategies (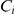) were calculated as follows:
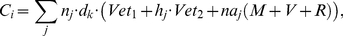
(2)where 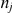 represents the number of farms of a specific type 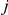 that should be vaccinated, 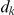 the number of doses that an animal type 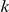 needs, 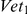 the standard call out charge and 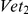 the hourly rate of an veterinarian. Hence, 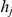 represents the number of hours needed to vaccinate all animals at farm 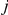, 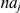 the number of animals to be vaccinated, 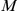 the costs for materials needed per animal, 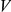 the costs of one dosage vaccine, and 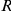 the costs for registration of the vaccinated animal.

The European Commission decided in 2008 to provide financial support to the emergency Bluetongue vaccination programmes of Member States. The financial contribution supported vaccination plans at a rate of 100% of the vaccine's supply cost and 50% of the costs incurred while carrying out the vaccination, up to certain ceilings [17]. The costs of the vaccination strategies adjusted for the EU compensation (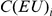) were calculated as follows:
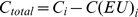
(3)where,
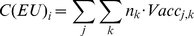
(4)In this equation, 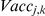 represents the vaccination costs per farm of type 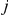 for animal types 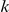, which were calculated based on the following ceilings as defined by the EU Commission [17]:
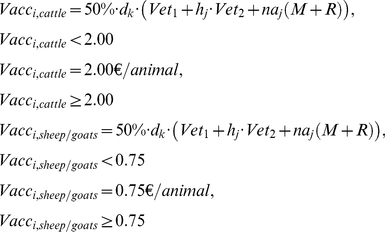
(5)


The inputs for the calculations are listed in Table 2. The demographic input on farm numbers and number of animals per farm originated from the Statistics Netherlands database [18]. The numbers on the cattle were based on the census conducted in May 2006 and the numbers related to sheep and goats on the census of November 2004. Based on the census data the number of animals present at a farm per year was estimated and used for the calculations. The cattle sector also included the export related firms, i.e. six export quarantine farms and 34 exporters (of which some have their own export quarantine farm). The numbers of sheep and hobby farms included also unregistered holdings, which are based on the estimation that 60% of the sheep holdings and 75% of the goat holdings were not registered [19]. All other inputs were based on expert opinions of either the vaccine producer or veterinarians.

### Economic criteria to rank the vaccination strategies

The different vaccination strategies are ranked bythe benefit-cost ratios or the total net returns. The benefit-cost ratio equals the total benefits divided by the total cost and shows how much benefits are generated at costs of one Euro. When it exceeds one, the strategy is economically efficient and the higher the ratio the better the efficiency. The net returns equal the total benefits minus the total costs. It shows how high the extra benefits are in relation to the total costs.

### Sensitivity analysis

A sensitivity analysis was performed to assess and identify the inputs that most influence the net benefits. Each individual input was changed with +10% and −10% and the total net benefit was calculated. This analysis was carried out using the add-in software TopRank 5.5 for Excel of Palisade Decision Tools [20].

## Results

### Financial impact of BT2008-NV epidemic

The financial impact of the BT2008-NV epidemic was estimated to be €40.9 and €41.3 mln for the epidemiological scenarios ES1 and ES2, respectively (Table 3). Most losses would be for the sheep breeding farms, the dairy export firms and the dairy farms being €12.6, €12.6 and €11.3 mln, respectively, whereas smallest losses could be expected for the goat farms, veal calf farms and sheep export firms. Looking at the different cost categories, it can be observed that the production losses resulted in the highest impact, 52,8% and 55,2% of the total net costs, respectively, followed by the financial impact of the expected control measures, i.e. 33.2% and 32.8%, respectively.

**Table 3:** Financial impact (* €1000) of the BT2008-NV epidemic according to farm type, sector and overall.

	Production losses		Diagnosis	Treatment		Control	Total	
Farm type/sector	ES1	ES2	-	ES1	ES2		ES1	ES2
**Cattle**								
Dairy	9,861.9	10,212.7	0.0	867.7	110.5	949.9	11,679.5	11,273.1
Veal calf	19.1	19.1	-	-	-	0.0	19.1	19.1
Other cattle	465.4	522.3	0.0	50.4	6.7	0.0	515.8	529.1
Susp test neg^1^	-	-	154.2	-	-	-	154.2	154.2
Screening	-	-	182.4	-	-	-	182.4	182.4
Export	-	-	-	-	-	12,576.0	12,576.0	12,576.0
**Cattle subtotal**	10,346.3	10,686.0	336.6	918.1	117.3	13,525.9	25,126.9	24,665.8
**Sheep**								
Dairy	11.1	11.1	0.0	4.4	4.4	0.0	15.6	15.6
Herding	138.5	138.5	0.2	198.2	198.2	0.0	337.0	337.0
Breeding	9,590.3	9,590.3	61.3	2,959.3	2,959.3	1.3	12,612.2	12,612.2
Fattening	1,000.0	1,000.0	11.8	871.0	871.0	0.5	1,883.3	1,883.3
Hobby	488.7	488.7	64.1	157.4	157.4	0.0	710.3	710.3
Susp test neg	-	-	91.7	-	-	-	91.7	91.7
Screening	-	-	26.1	-	-	-	26.1	26.1
Export	-	-	-	-	-	1.8	1.8	1.8
**Sheep subtotal**	11,228.7	11,228.7	255.3	4,190.3	4,190.3	3.6	15,677.9	15,677.9
**Goats**								
Dairy	0.4	0.4	0.6	0.6	0.6	-	1.6	1.6
Fattening	0.0	0.0	0.0	0.0	0.0	-	0.0	0.0
Hobby	0.0	0.0	0.8	0.2	0.2	-	1.0	1.0
Susp test neg	-	-	3.3	-	-	-	3.3	3.3
Screening	-	-	26.1	-	-	-	26.1	26.1
Export	-	-	-	-	-	18.2	18.2	18.2
**Goat sector subtotal**	0.4	0.4	30.8	0.8	0.8	18.2	50.2	50.2
**Total**	21,575.5	22,811.6	622.6	5,109.3	4,308.4	13,547.7	40,855.1	41,290.3

1 Suspected farm but tested negative.

### Benefits of vaccination strategies

**Figure 1:**
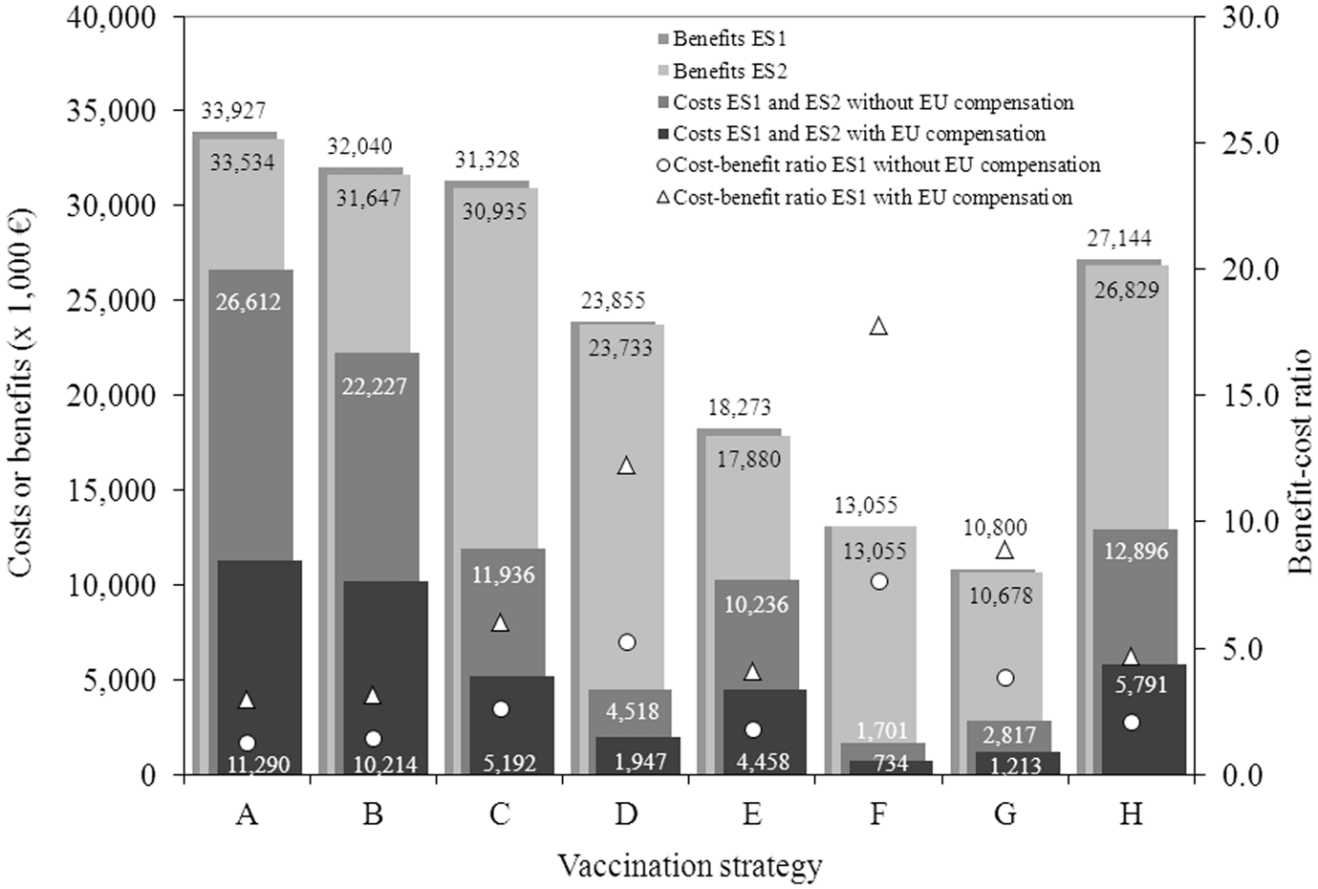
Benefits, costs and cost-benefit ratios of the eight vaccination strategies for the two epidemiological scenarios 1 and 2 (Sc1 and Sc2) where the strategies are ranked descending according to the net benefits of scenario 2.

Most benefits (i.e. reduction in losses compared to the BT2008-NV epidemic) are gained with strategies A, B and C, which are about €33.9, €32.0 and €31.3 mln, respectively (Figure 1). Whereas, strategy G brings the least benefits (€10.8 mln) followed by strategy F (€13.1 mln). The difference between the two epidemiological scenarios ES1 and ES2 is relatively small, i.e. this difference varies between €0 and €393. Therefore we will show only the results based on ES1 in the remainder of this paper.

### Vaccination costs

The costs of the vaccination strategies varied widely. Strategy G costs only €2.8 mln or €1.2 mln, with or without EU compensations respectively, whereas strategy A costs almost 10 times more, i.e. €26.6 mln or €11.3 mln (Figure 1). Vaccination costs differed among farm types (Table 4). To vaccinate an average dairy farm the costs sum up to €383 per farm if all animals should be vaccinated or €333 if only adults should be vaccinated, whereas vaccination of a dairy sheep farm costs €250 to €252 and of a dairy goat farm €347 to €425.

**Table 4:** Vaccination costs per sector (*1,000), farm and animal, the percentage financed by the EU and the percentage of the costs for the Veterinary labour.

	Vaccination costs (€)						% of the Vaccination costs			
	Per (sub) sector		Per farm		Per animal^1^		Financed by EU		Veterinary labour	
Farm type	All	Only	All	Only	All	Only	All	Only	All	Only
	Animals^2^	Adults^3^	animals	adults	animals	adults	animals	adults	animals	adults
**Cattle**										
Dairy	8,540.2	7,433.8	383	333	3.29	5.24	61%	38%	36%	41%
Veal calf	2,002.2	0.0	631	0	4.74	-	42%	-	40%	-
Other cattle	2,995.5	2,801.9	308	288	8.39	18.56	24%	11%	44%	47%
**Sector total**	13,537.8	10,235.7								
**Sheep**										
Dairy	7.6	7.5	252	250	1.03	1.04	70%	70%	54%	55%
Herding	48.0	18.5	1,200	463	0.60	1.03	85%	70%	21%	55%
Breeding	2,149.4	1,674.7	206	161	1.40	3.17	54%	24%	66%	85%
Fattening	420.9	0.0	210	0	1.34	-	56%	-	65%	-
Hobby	4,272.1	4,183.8	82	81	10.50	19.12	7%	4%	96%	98%
**Sector total**	6,898.0	5,884.5								
**Goats**										
Dairy	149.0	121.7	425	347	0.69	0.78	80%	77%	32%	39%
Fattening	12.4	0.0	276	0	0.93	-	73%	-	49%	-
Hobby	6,014.5	5,985.6	80	80	21.93	28.12	3%	3%	98%	98%
**Sector total**	6,175.9	6,107.4								

1 Per vaccinated animal.

2 These number equal vaccination strategy A.

3 These numbers equal vaccination strategy B.

The vaccination costs are almost twice as high for the cattle sector as for the sheep and goat sectors (Table 4). This is mainly because cattle must be vaccinated twice and sheep and goats only once. If only adults are vaccinated, it saves €3.3 mln vaccination costs in the cattle sector and €1.0 mln in the sheep sector, whereas only €0.1 mln in the goat sector. The vaccination costs at farm level vary widely due to economies of scales where the costs per animal are lower for larger farms than for smaller farms, although larger farms have higher total cost. Compare for example, the cost per farm or animal for the sheep breeding farms (€206 per farm and €1.40 per animal) with the sheep hobby farms (€82 per farm and €10.50 per animal). The relative size of the EU compensation for the vaccination programmes varies much between farm types, i.e. between 3% and 85% per farm type. This variation is caused by the difference in farm size, but also by the ceilings per animal that are configured by the Commission. The labour costs for the veterinarian are relatively high (varying between 21% and 98%). This is because only a veterinarian is allowed to vaccinate the animals in the Netherlands and labour costs in general are high compared to other cost components.

### Costs compared to the benefits

**Figure 2:**
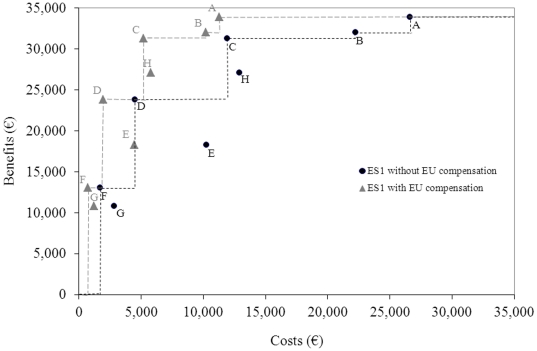
Least cost - highest benefit frontier for the eight vaccination strategies.

In Figure 2, the benefits of the vaccination strategies are plotted against the corresponding vaccination costs. The dashed lines represents the least cost - highest benefit frontiers for the ES1 scenario, with and without EU compensation respectively. The points located on the frontier are considered as the most efficient set of strategies, whereas the points below the frontier as inefficient. For each strategy below the frontier, there is at least one strategy on the frontier that has either lower costs and/or more benefits or both. The least-cost frontier is excluding strategies G, E and H, indicating that these are less efficient than others.

The overall benefit-cost ratios (for the three sectors together) are for all vaccination strategies efficient, i.e. higher than one (Table 5). The highest benefit-cost ratio is expected for scenario F (7.68), followed by strategy D (5.28). Strategy G has the third best benefit-cost ratio (3.83), but is less efficient than F and D according to the least cost – highest benefit frontier. The benefit-cost ratios for the goat sector are low and are, therefore, not efficient suggesting that vaccinating goats next to sheep or cattle is from an economic point of view not sensible. The benefit-cost ratios of the sheep and cattle sectors exceed one for all strategies, where the ratios of the sheep sector are for all vaccination strategies higher than the cattle sector ratios.

**Table 5:** Estimated benefit-cost ratios of the eight defined BTV-8 vaccination strategies for different farm types, for the three sectors and for all sectors overall.

	*Benefi- cost ratios of the different vaccination strategy (A–H)*							
Farm type/sector	A	B	C	D	(E)^a^	F	(G)	(H)
Dairy	1.17	1.35	1.35	1.35	1.35	-	1.35	1.35
Veal calf	0.01	∞	∞	∞	∞	-	∞	∞
Other cattle	0.17	0.18	0.18	0.18	0.18	-	0.18	0.18
Export related	0.00	∞	∞	∞	∞	-	∞	∞
**Cattle sector**	**1.35**	**1.79**	**1.79**	**3.83**	**1.79**	**-**	**3.83**	**1.97**
Dairy sheep	2.06	2.08	2.08	2.08	-	2.08	-	2.08
Traditional herding	7.02	18.18	18.18	18.18	-	18.18	-	18.18
Breeding	5.87	7.53	7.53	7.53	-	7.53	-	7.53
Fattening	4.47	-	-	-	-	-	-	-
Hobby	0.17	0.17	-	-	-	-	-	0.17
**Sheep sector**	**2.27**	**2.34**	**7.68**	**7.68**	**-**	**7.68**	**-**	**2.34**
Dairy goat	0.01	0.01	-	-	-	-	-	-
Fattening	0.00	-	-	-	-	-	-	-
Hobby	0.00	-	-	-	-	-	-	-
**Goat sector**	**0.00**	**0.00**	**-**	**-**	**-**	**-**	**-**	**-**
**All sectors**	**1.27**	**1.44**	**2.62**	**5.28**	**1.79**	**7.68**	**3.83**	**2.10**
**Ranking**	**8**	**7**	**4**	**2**	**(6)**	**1**	**(3)**	**(5)**

a The strategies between parentheses are less efficient than others: see least cost frontier of Figure 2.

Not all farm types have efficient ratios (Table 5). The ‘other cattle farms’, the ‘sheep hobby farms’ and all goat farms have benefit-cost ratios lower than one, suggesting that it might be better to exclude them from a vaccination strategy from an economic point of view. Moreover, some farms/holdings have infinitive benefit-cost ratios. These are the veal calf farms and the export related cattle farms for the strategies where they do not have to vaccinate (no vaccination costs) but where they have economic benefits: some for the veal calf farms and extensive farms benefits for the export related cattle firms.

The net returns (for the three sectors together) have a positive value for all vaccination strategies indicating that applying a vaccination strategy is better than doing nothing (Table 6). The highest net returns are expected for strategies C and D (i.e. about €19 mln) followed by strategy H (i.e. around €14 mln), although strategy H is less efficient than others according to the least-cost frontier.

**Table 6:** Net returns of the eight defined BTV-8 vaccination strategies for different farm types, for the three sectors and for all sectors together.

	Net returns (* €1,000) of the different vaccination strategy (A–H)							
Farm type/sector	A	B	C	D	(E)^a^	F	(G)	(H)
Dairy	1,477	2,583	2,583	795	2,583	0	795	2,067
Veal calf	−1,983	19	19	6	19	0	6	15
Other cattle	−2,480	−2,286	−2,286	−432	−2,286	0	−432	−1,829
Export related	7,567	7,567	7,567	7,567	7,567	0	7,567	7,567
**Cattle sector**	**4,735**	**8,037**	**8,037**	**7,983**	**8,037**	**0**	**7,983**	**7,943**
Dairy sheep	8	8	8	8	0	8	0	6
Traditional herding	289	318	318	318	0	318	0	255
Breeding	10,462	10,936	10,936	10,936	0	10,936	0	8,749
Fattening	1,462	0	0	0	0	0	0	0
Hobby	−3,562	−3,473	0	0	0	0	0	−2,779
**Sheep sector**	**8,750**	**7,881**	**11,354**	**11,354**	**0**	**11,354**	**0**	**6,305**
Dairy goat	−147	−120	0	0	0	0	0	0
Fattening	−12	0	0	0	0	0	0	0
Hobby	−6,014	−5,986	0	0	0	0	0	0
**Goat sector**	**−6,170**	**−6,106**	**0**	**0**	**0**	**0**	**0**	**0**
**All sectors**	**7,315**	**9,812**	**19,391**	**19,337**	**8,037**	**11,354**	**7,983**	**14,248**
**Ranking**	**8**	**5**	**1**	**2**	**(6)**	**4**	**(7)**	**(3)**

a The strategies between parentheses are less efficient than others: see least cost frontier of Figure 2.

When only adult cattle are vaccinated, the net returns increases by at least €2.5 mln (e.g. comparing strategy A with B). This indicates that it is not cost efficient to vaccinate young stock. This is because the vaccination costs increases by €4.4 mln if young animals in the cattle, sheep and goat sectors would also be vaccinated, whereas the reduction in losses is only 1.8 mln Euros (comparing strategy A with B). The limited reduction in losses is due to the fact that most production losses are related to adult animals and moreover most diseased animals were adults [11]. Excluding hobby farms (and the category “other cattle” farms) from a vaccination strategy would increase the net returns of the sectors with about €2.3 million for cattle and €3.5 million for sheep.

Summarizing, strategy D (vaccination of all adult sheep at professional farms of the Netherlands and all adult cattle in the four Northern provinces) is the best strategy to apply based on economic criteria. Strategies C (vaccination of all adult sheep and cattle at professional farms in the Netherlands) and F (vaccination of all adult sheep at professional farms in the Netherlands) are the second and third best strategies. The strategies E (vaccination of all adult cattle at professional farms in the Netherlands), G (vaccination of all adult cattle at professional farms in the four Northern provinces) and H (vaccination of 80% of the adult sheep and cattle in the Netherlands) have more efficient alternatives and should therefore not be considered. Whereas the strategies A (vaccination of all sheep, cattle and goats of the Netherlands) and (to a less extend) B (vaccination of all adult sheep, cattle and goats of the Netherlands) are ranked last.

### Sensitivity analysis

**Figure 3:**
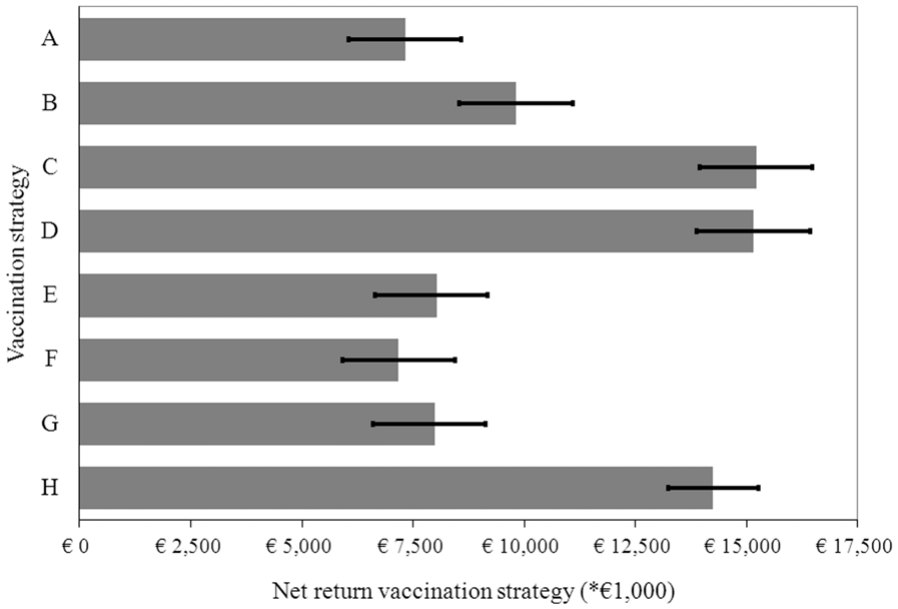
The maximal difference in net return caused by the most influential input when changing it with 10%.

The effect of individual inputs on the total net returns for the different vaccination strategies is limited (Figure 3). The number of animals per farm and the number of farms in the Netherlands were most influential. The time needed to vaccinate a farm is one of the most influential inputs, although it affects the net returns little.

## Discussion

The objective of this study was to rank eight vaccination strategies based on efficiency (i.e. net cost in relation to prevented losses or benefits) for controlling the BTV-8 epidemic in 2008 in the dairy, sheep and goat sectors. This was done using an economic model that includes the Dutch professional cattle, sheep and goat sectors together with the hobby farms. All financial costs and benefits (that can be linked to the BTV-8 epidemic and/or the control measures) of a wider farm perspective, including the affected farm sector (related to the vaccination strategies in the cattle, sheep and goats), the affected related industry and the controlling authorities were included, regardless of who is paying. It can be concluded that strategies F (where all adult sheep at professional farms are vaccinated) and C (where all adult sheep and cattle at professional farms are vaccinated) are the best strategies based on the benefit-cost ratio and that C and D (where all adult sheep at professional farms and all adult cattle in the four Northern provinces are vaccinated) are best based on the net return.

Hence the decision of the vaccination strategies depends on the economic criteria used, but the top three efficient strategies include the same strategies, namely C, D and F. Which criteria is best is subject to the preference of the decision maker. The cost-benefit ratio where costs are divided by the benefits is a good indicator for the return on investment, but if the investment is low the ratio can be most efficient if the benefits are relatively high compared to the costs. The net return where the difference between the costs and benefits are calculated indicates how high the returns are [21]. Both indicators are used in decision making and which one to prefer is mostly based on the preference of the decision maker.

Epidemiological assumptions had to be made as at the time of this study not all epidemiological knowledge was available. One of the assumptions was that all farms that were not infected in 2007 would be infected in the 2008 epidemic if no vaccination strategy would be applied. As we now know that BT virus dynamics are predominantly driven by environmental conditions (e.g. temperature, and vector habitats) [9] but might also be driven by housing systems or other factors this is a questionable assumption. So the losses prevented in all vaccination scenarios could be overestimated. However, the ranking of the strategies would stay the same in case of a reduced infection rate. Also, new mortality rates have been reported for the Netherlands suggesting that the mortality in scenario B was underestimated [22], suggesting that our estimation of the prevented losses was underestimated. Again, we think that the ranking would not be affected, as the underestimations applies to all vaccination scenarios. Another assumption made is that the presence of vaccinated animals in an area will not result in a benefit for other non-vaccinated animals, due to a reduced transmission rate. This assumption was made as a lot knowledge about BTV8 transmission was unknown at the time of this study. This assumption might lead to an underestimation of the losses prevented for scenarios where some animal groups were excluded. The efficiency of vaccination strategy D might therefore be underestimated compared to strategy C.

We assumed that animals were vaccinated a few weeks before being exposed to BTV-8 so that the effect of vaccination was maximal. At the time of this study it was a hard assumption, as the vaccine was available at the start of June 2008 and the first outbreaks in previous 2006 and 2007 were observed at the start of August and at the end of July: leaving 8 weeks to vaccinate the population. However, the logistic plan behind the vaccination campaign was ready at the time of this study and showed that most animals could be vaccinated within a short time frame.

This study included a time horizon of only one year and excluded therefore the longer term benefits of eradicating BTV-8 from the Netherlands and the EU. Including a longer time horizon would include other effects like eradication and reestablishment of the export markets, but epidemiological knowledge regarding the developments in the future was at that time missing. For example, are naturally infected animals and vaccinated animals protected against BTV-8 for the rest of their lives or only for a shorter period? How long would maternally gained immunity proceed? How long will it take before eradication is achieved in the Netherlands, the neighbouring countries (Belgium and Germany) and the whole of the EU?

This study included all affected sectors: cattle, sheep and goats. Not only the primary producers were considered, but also other chain partners, like slaughterhouses or dairy firms. However, after the first round of discussions with sector experts, no negative or positive effects of the BTV-8 epidemic of these firms were indicated, which was confirmed by the statistical analysis of product prices. The effect of vaccination on the consumption of animal products was also expected to be zero. Note that the analysis of the price changes was based on the monthly average national prices of the different animals and animal products. To check for missed price changes and/or changes that were caused by other factors market experts were asked to give their expert opinion. This analysis provided us an idea whether prices change at National level but not for the farms within a restriction zone compared to outside a restriction zone. As the analysis is focuses on sectors the latter is less important. The economic benefits of the veterinarians due the BTV-8 epidemic were not quantified explicitly. But the benefits can be high when looking to the veterinary labour costs relatively to the total vaccination costs.

The results show that it is not cost efficient to vaccinate goats. Not a lot of severe clinical symptoms [23] and consequently negative production effects were observed in the goat populations during the BTV-8 epidemic. Furthermore, there is no economic important export market for the goat sector that should be maintained. Therefore, almost no benefits are expected for this sector if vaccination would be applied here, resulting in an benefit-cost ratio lower than one and a negative net return.

Vaccinating hobby farms is not cost efficient in a time horizon of a year. This is mainly because the vaccination costs are relatively high (per farm or animal) due to the high costs for the veterinarian. Based on economic reasoning, excluding the hobby farms for a vaccination strategy is for this reason sensible. However, note that the importance of economic criteria for hobby farmers is less than for commercial farms so giving them the possibility to vaccinate for animal welfare reasons would be fair.

### Epilogue

Shortly after finishing this study the Dutch Government had to decide on the strategy to implement. They decided to apply a voluntary vaccination program aiming a vaccination coverage of 80% in order to get the financial support from the EU. The 80% coverage rule of the EU was based on the assumption that 80% coverage would be needed to prevent between-herd transmission and that eradication might become feasible. The UK (England and Wales) also applied a voluntary program, whereas other countries applied mandatory vaccination programs (Belgium, Germany, Luxembourg and the Czech Republic).

The estimated vaccination coverage varied from one country to the other. In the Netherlands the estimated vaccination coverage was: 73% in sheep, 71% in dairy farms, 43% in goat farms and 67% in hobby holdings [24]. In the UK a coverage of >80% was achieved within areas where BTV transmission has been confirmed in 2007, but it was about 40% in areas where no BTV had been reported before. In Germany, approximately 70% of cattle and 90% of sheep in the infected areas were vaccinated [25].

Again a voluntary vaccination program was implemented in the Netherlands in 2009, although now no financial support by the EU was available. The willingness of Dutch farmers to vaccinate was significantly lower that year, namely an estimated 42% in sheep farms, 58% in dairy farms, 19% in goat farms and 49% in hobby farms [24]. No new BTV-8 infections were reported in that year [26].

A voluntary program as implemented in the Netherlands in 2008 might be less efficient as other compulsory strategies. However, other than economic criteria are used in making the decision on a vaccination strategy. Mandatory strategies must first be approved by the EU and may carry additional administrative requirements. This, in combination with delay that may result from mandatory vaccination strategies is the main reason why not every country choices for a mandatory strategy. Furthermore, the Dutch government imposes increasingly the responsibility for disease outbreaks back to the industry. And, additionally the negative attitude of farmers against compulsory vaccination is partly caused by the negative experience they had with a BVDV-contaminated BHV type 2 vaccine used in an earlier compulsory vaccination campaign [24], [27]. This contaminated vaccine caused health problems with cattle , resulting in a negative experience that still triggers some distrust with respect to vaccination campaigns in Dutch cattle farmers [24].
